# Testing for Sufficient-Cause Interactions in Case-Control Studies of Non-Rare Diseases

**DOI:** 10.1038/s41598-018-27660-2

**Published:** 2018-06-18

**Authors:** Jui-Hsiang Lin, Wen-Chung Lee

**Affiliations:** Institute of Epidemiology and Preventive Medicine, College of Public Health, National Taiwan University, Taipei, Taiwan

## Abstract

Sufficient-cause interaction (also called mechanistic interaction or causal co-action) has received considerable attention recently. Two statistical tests, the ‘relative excess risk due to interaction’ (RERI) test and the ‘peril ratio index of synergy based on multiplicativity’ (PRISM) test, were developed specifically to test such an interaction in cohort studies. In addition, these two tests can be applied in case–control studies for rare diseases but are not valid for non-rare diseases. In this study, we proposed a method to incorporate the information of disease prevalence to estimate the perils of particular diseases. Moreover, we adopted the PRISM test to assess the sufficient-cause interaction in case–control studies for non-rare diseases. The Monte Carlo simulation showed that our proposed method can maintain reasonably accurate type I error rates in all situations. Its powers are comparable to the odds-scale PRISM test and far greater than the risk-scale RERI test and the odds-scale RERI test. In light of its desirable statistical properties, we recommend using the proposed method to test for sufficient-cause interactions between two binary exposures in case–control studies.

## Introduction

The assessment of interactions is a critical issue in epidemiology. Recently, a particular kind of interaction, the sufficient-cause interaction (also called mechanistic interaction or causal co-action), has received much attention^1^. Two statistical tests, the ‘relative excess risk due to interaction’ (RERI) test^1–5^ and the ‘peril ratio index of synergy based on multiplicativity’ (PRISM) test^6^, were developed specifically to test such an interaction. A RERI test is based on risk additivity, and a PRISM test is based on log peril additivity, where peril is defined to be the inverse of a survival. Risks and perils should be estimated in a cohort study; therefore, both tests are to be used in such a study.

Previously, Lee^6,7^ and Lin and Lee^8^ mentioned that risks and log perils can be approximated by odds ratios under the rare-disease assumption. The above two tests then become a RERI test on the odds ratio scale, and therefore can be used in a case–control study^6–11^. (Odds ratios are readily estimable in a case–control study.) However, the approximation would break down for non-rare diseases. Lin and Lee^8^ showed that risks and perils cannot be estimated in case–control studies unless the sampling fractions of cases and controls are known; however, researchers rarely have this information. At present, neither RERI nor PRISM tests can be valid for sufficient-cause interaction in case–control studies for non-rare diseases^6–11^.

In this study, we proposed a method to incorporate the information of disease prevalence to estimate disease perils. Then, we adopted a PRISM test to assess the sufficient-cause interaction in a case–control study for non-rare diseases^6^. We examined the statistical properties of the proposed method using a Monte Carlo simulation and demonstrated its use on real data.

## Method

We evaluated the sufficient-cause interaction between two binary exposures ($X$ and $Z$) and a binary outcome. In a cohort study of a population within a certain time interval, $(0,T)$, we used a PRISM test to assess the sufficient-cause interaction proposed by Lee^6^. Here, we used the same notations as in the previous studies^6–8^. For people in the population with exposure profiles of $X=x$ and $Z=z$ for $x,z\in \{0,1\}$, ${{\rm{Risk}}}_{x,z}$ denoted the disease risk in $(0,T)$; ${{\rm{Odds}}}_{x,z}$, the disease odds in $(0,T)$; and ${{\rm{Peril}}}_{x,z}={(1-{{\rm{Risk}}}_{x,z})}^{-1}$, the disease peril in $(0,T)$. We calculated ${\rm{logPRISM}}={{\rm{logPeril}}}_{1,1}-{{\rm{logPeril}}}_{1,0}-{{\rm{logPeril}}}_{0,1}+{{\rm{logPeril}}}_{0,0}$, and sufficient-cause interactions were declared when logPRISM was statistically different from zero^6^.

Assuming that a case–control study recruited a total of ${N}_{1}$ cases and ${N}_{0}$ controls, ${\hat{q}}_{x,z}$ denoted the sample proportion of subjects with an exposure profile of $X=x$ and $Z=z$ recruited in the case group and ${\hat{r}}_{x,z}$ in the control group. Because disease perils cannot be estimated in a case–control study, a PRISM test cannot be applied directly in such a study^6–8^. Therefore, we proposed a method to estimate disease perils in a case–control study. First, we required an estimate of the overall disease prevalence $\hat{p}=\frac{D}{N}$ of the study population from vital statistics, where $N$ denoted the population size and $D$ denoted the total number of the diseased subjects. According to Bayes’ theorem^1,12–14^, we could then estimate the log perils as $\mathrm{log}\,{\widehat{{\rm{Peril}}}}_{x,z}=\,\mathrm{log}(1+{\widehat{{\rm{Odds}}}}_{x,z})=\,\mathrm{log}(1+\frac{\hat{p}}{1-\hat{p}}\times \frac{{\hat{q}}_{x,z}}{{\hat{r}}_{x,z}})$. Note that here an estimate of the overall disease prevalence ($\hat{p}$) suffices. There is no need to further obtain sex, age, or exposure profile-specific disease prevalence.

Next, we calculate

$$\begin{array}{rcl}\mathrm{log}\,\widehat{{\rm{PRISM}}} & = & \mathrm{log}(1+\frac{\hat{p}}{1-\hat{p}}\times \frac{{\hat{q}}_{1,1}}{{\hat{r}}_{1,1}})-\,\mathrm{log}(1+\frac{\hat{p}}{1-\hat{p}}\times \frac{{\hat{q}}_{1,0}}{{\hat{r}}_{1,0}})\\  &  & -\,\mathrm{log}(1+\frac{\hat{p}}{1-\hat{p}}\times \frac{{\hat{q}}_{0,1}}{{\hat{r}}_{0,1}})+\,\mathrm{log}(1+\frac{\hat{p}}{1-\hat{p}}\times \frac{{\hat{q}}_{0,0}}{{\hat{r}}_{0,0}}).\end{array}$$

The PRISM test is a Z-test: $Z=\frac{\mathrm{log}\,\widehat{{\rm{PRISM}}}}{\sqrt{{\rm{Var}}(\mathrm{log}\,\widehat{{\rm{PRISM}}})}}$, where ${\rm{Var}}(\mathrm{log}\,\widehat{{\rm{PRISM}}})$ is detailed in S1 Exhibit. Sufficient-cause interactions can be declared when the test statistics $Z$ is in the rejection region (for the null hypothesis of no sufficient-cause interaction). R code (S2 Exhibit) and SAS code (S3 Exhibit) are provided for all computations.

### Simulation studies

**Figure 1:**
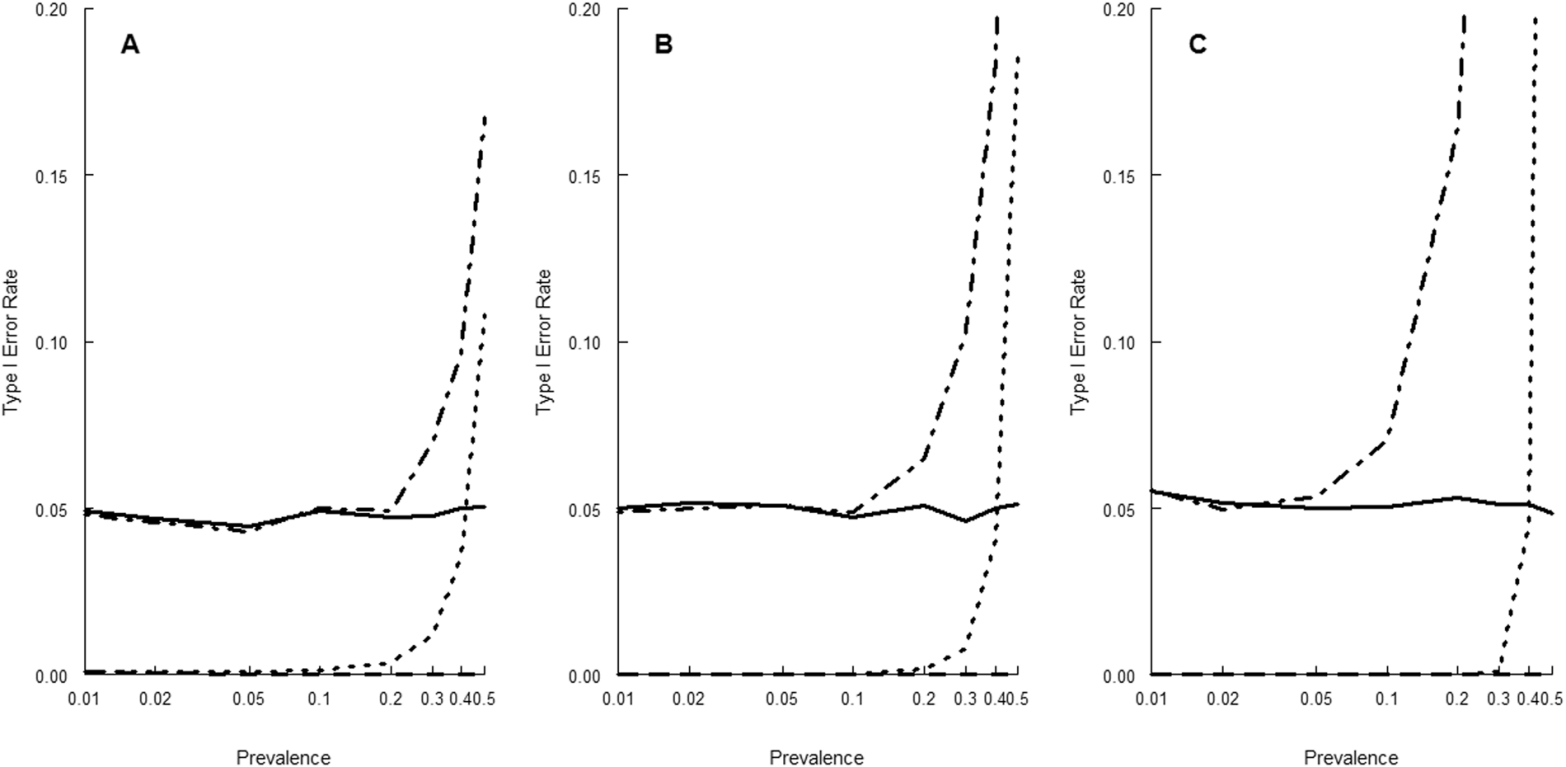
Type I error rates under the null hypothesis of no sufficient-cause interaction: 500 cases and 500 controls (**A**), 1000 cases and 1000 controls (**B**), and 5000 cases and 5000 controls (**C**). Solid lines are the type I error rates for the proposed method, dashed lines, those for the risk-scale RERI test, dotted lines, those for the odds-scale RERI test, and dashdotted lines, those for the odds-scale PRISM test.

**Figure 2:**
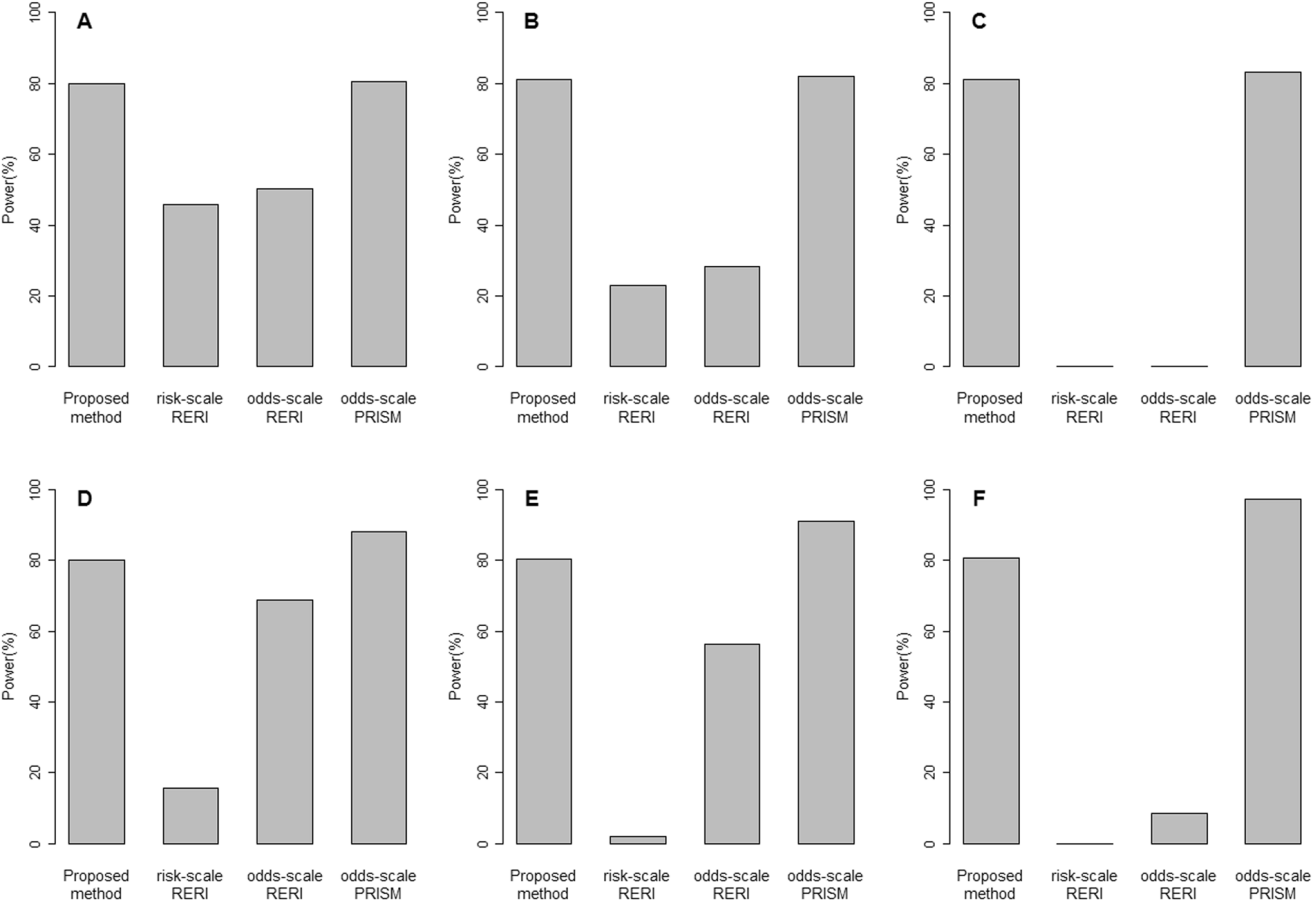
The powers under the alternative hypothesis, respectively, when the disease prevalence is 0.02 (upper panel) and when it is 0.2 (lower panel): 500 cases and 500 controls (**A**,**D**), 1000 cases and 1000 controls (**B**,**E**), and 5000 cases and 5000 controls (**C**,**F**).

A Monte Carlo simulation was conducted to evaluate the proposed method. We assumed that in the study population, the prevalence values of both $X$ and $Z$ were 0.5, the relative risk for $X$ was ${{\rm{RR}}}_{1,0}=3$ and the relative risk for $Z$ was ${{\rm{RR}}}_{0,1}=2$, where ${{\rm{RR}}}_{x,z}=\frac{{{\rm{Risk}}}_{x,z}}{{{\rm{Risk}}}_{0,0}}$. We considered different sample sizes and assumed that a case–control study recruited 500 cases and 500 controls (Panel A in Fig. 1, and Panels A and D in Fig. 2), 1000 cases and 1000 controls (Panel B in Fig. 1, and Panels B and E in Fig. 2), and 5000 cases and 5000 controls (Panel C in Fig. 1, and Panels C and F in Fig. 2), respectively. We checked the type I error rate under the null hypothesis of no sufficient-cause interaction (${\rm{PRISM}}=1$) when the disease prevalence was 0.01, 0.02, 0.05, 0.1, 0.2, 0.3, 0.4, and 0.5, respectively. We examined the powers under the alternative hypothesis, respectively, when the disease prevalence was 0.02 (${\rm{PRISM}}=1.0155$, $1.0109$, and $1.0048$, for Panels A, B, and C in Fig. 2, respectively) and when it was 0.2 (${\rm{PRISM}}=1.1710$, $1.1172$, and $1.0500$ for Panels D, E, and F in Fig. 2, respectively). We assumed that the estimates of the disease prevalence were derived from the vital statistics with the population size $N={10}^{6}$. A total of 10,000 simulations were performed for each scenario. The level of significance was set at $\alpha =0.05$.

For comparison, we also performed a risk-scale RERI test, an odds-scale RERI test, and an odds-scale PRISM test. For the risk-scale RERI test, we incorporated $\hat{p}$ from vital statistics to the case–control study to estimate the disease risks necessary for calculating RERI, similar to what we did to estimate the disease perils necessary for calculating PRISM (more details in S4 Exhibit). Both the odds-scale RERI test and the odds-scale PRISM test are the approximation mentioned in the previous studies^6–11^. For the odds-scale RERI test, we used odds ratios to approximate relative risks^7–9^. For the odds-scale PRISM test, we used the approximation^6–8^:

$${\rm{logPRISM}}\approx {{\rm{Odds}}}_{1,1}-{{\rm{Odds}}}_{1,0}-{{\rm{Odds}}}_{0,1}+{{\rm{Odds}}}_{0,0}.$$

Figure 1 shows the type I error rates. For the proposed method, the type I error rates are very close to the nominal $\alpha$ level for all scenarios. For the odds-scale PRISM test, type I error rates are stable at 0.05 at low disease prevalence but are inflated when the disease prevalence is greater than 0.2. With a larger sample size, the type I error rates for the odds-scale PRISM test are inflated even at low disease prevalence values. By contrast, the risk-scale RERI test is a very conservative test with extremely small type I error rates. As for the odds-scale RERI test, its type I error rates are small at low disease prevalence values but can become inflated when the disease prevalence is greater than 0.4.

Figure 2 shows the simulation results of the powers. The powers of the proposed method reached more than 80% in all scenarios. The powers of the odds-scale PRISM test are comparable to (when the disease prevalence is 0.02) and greater than (when the disease prevalence is greater than 0.2) those of the proposed method. However, we should note that the type I error rates of the odds-scale PRISM test are inflated when the disease prevalence is 0.2. The risk-scale RERI test and the odds-scale RERI test are much less powered compared with the proposed method. Table 1 summarized the comparative results of four methods.

**Table 1:** A summary of the simulation results

	Proposed method	Risk-scale RERI test	Odds-scale RERI test	Odds-scale PRISM test
Type I error rate	Stable at 0.05 for all scenarios.	Extremely small, very conservative test.	Small at low disease prevalence values, but inflated when the disease prevalence is greater than 0.4.	Stable at 0.05 at low disease prevalence, but inflated even at low disease prevalence values with larger sample sizes.
Power	Reached more than 80% in all scenarios.	Much less powered compared with the proposed method	Much less powered compared with the proposed method	Comparable to (when the disease prevalence is 0.02) and greater than (when the disease prevalence is greater than 0.2) those of the proposed method.

The proposed method also reveals the desirable statistical properties in further simulation with unbalanced sample sizes between the case and control groups and unequal prevalence between two exposures.

### An Example

We used Tong *et al*.’s^15^ case–control data on essential hypertension to demonstrate our method. The case–control study assessed the effects of *A1166C* site of *AT1R* gene polymorphism (*AC*+*CC* versus *AA* genotypes) and noise exposure ($\ge 85{\rm{dB}}$ versus <85 dB) on essential hypertension (see Table 2). Based on a multiplicative model, Tong *et al*.^15^ concluded that gene-noise *multiplicative* interaction may play a role for essential hypertension.

**Table 2:** Testing for sufficient-cause interaction in a case–control study on essential hypertension.

Genotype	Noise Exposure	Essential Hypertension		${\bf{log}}\,\widehat{{\bf{Peril}}}$
		Case	Control	
*AC*+*CC*	$\ge$85 dB	20	18	0.559
*AC*+*CC*	$<$85 dB	13	24	0.311
*AA*	$\ge$85 dB	161	261	0.348
*AA*	$<$85 dB	117	319	0.221

$$\mathrm{log}\,\widehat{{\rm{PRISM}}}(95 \% {\rm{CI}})=0.121(\,-\,0.214,0.456)$$.

To use our method, we need an estimate of disease prevalence for the study population. Tong *et al*. mentioned in their paper^15^ that the hypertension prevalence is 25.2% with a population size of 100,000. With this information and using the method presented in this paper, we calculated the log perils for the four exposure profile (Table 2). For this example, the logPRISM is 0.121 with a 95% confidence intervals of $(-0.214,0.456)$ and a *P* value of 0.478. We therefore conclude that there is no gene-noise *sufficient-cause* interaction on essential hypertension.

## Discussion

We proposed a method to incorporate information regarding disease prevalence to estimate disease perils, and then adopted a PRISM test to assess the sufficient-cause interaction in a case–control study. In our method, only the disease prevalence of the population at large is required ($\hat{p}$) and not the detailed sex, age, or exposure profile-specific prevalence; the overall prevalence is readily available from vital statistics or previously published studies. For non-rare diseases, we showed that the odds-scale RERI test and the odds-scale PRISM test (where risks and log perils are approximated by odds directly) tend to become too liberal. Furthermore, we showed that the external information (regarding the overall disease prevalence) need not be exact (S5 Exhibit). Researchers can comfortably apply the disease prevalence estimated from a study with sample size as small as 1000 without excessively impairing the power. For rare diseases, the odds-scale PRISM test is comparable to the proposed method; however, it is not applicable (the approximation breaks down) at lower prevalence with a larger sample size. We recommended using the proposed method for its reliable performance in all situations.

In a case–control study, the odds ratio is readily estimable to admit inferences about exposure–disease associations. Inferences can also be made using the risk ratio scale by invoking the rare-disease assumption.^1^ For non-rare diseases, Cornfield and other researchers^12–14^ noted that if an estimate of the disease prevalence of the study population at large is available, then absolute disease risks/odds for each exposure profile can be estimated. We followed Cornfield’s logic to incorporate the overall disease prevalence to estimate absolute disease risks/perils for each exposure profile. Therefore, the proposed method can be valid in assessing sufficient-cause interaction in case–control studies for non-rare diseases. Also, it can maintain reasonably accurate type I error rates. Its powers are comparable to those of the odds-scale PRISM test and far greater than those of the risk-scale RERI test and the odds-scale RERI test.

In conclusion, in light of its desirable statistical properties, we recommend using the proposed method to test for sufficient-cause interactions between two binary exposures in case–control studies. Further work is warranted to cast the proposed method in a general regression framework.

## Electronic supplementary material

Supplementary information
