# Supplementary material for: Testing for Sufficient-Cause Interactions in Case-Control Studies of Non-Rare Diseases
Source: Sci Rep. 2018 Jun 18;8:9274. doi: 10.1038/s41598-018-27660-2 (PMC6006284; doi:10.1038/s41598-018-27660-2)
Supplement: Supplementary file 1 — Supplementary information [file 41598_2018_27660_MOESM1_ESM.pdf]

Title: Testing for Sufficient-Cause Interactions in Case-Control Studies of Non-Rare Diseases

Authors: Jui-Hsiang Lin, Wen-Chung Lee

Supplementary information:

S1 Exhibit. The algorithm of the variance of logPRISM in the proposed method.

S2 Exhibit. R code for the proposed method.

S3 Exhibit. SAS code for the proposed method.

S4 Exhibit. The algorithm of the variance in the risk-scale RERI test

S5 Exhibit. The powers of the proposed method when incorporating the disease prevalence from a previous study with different sample size.

S1 Exhibit. The algorithm of the variance of logPRISM in the proposed method.

Let $\hat{\mathbf{P}}$ denote a 2×2 matrix with all its elements being $\frac{\hat{p}}{1-\hat{p}}$, and $\mathrm{Var}\hat{\mathbf{P}}$, a 4×4 matrix with all its elements being $\left(\frac{\hat{p}}{1-\hat{p}}\right)^2\times\left(\frac{1}{D}+\frac{1}{N-D}\right)$, the variance of $\frac{\hat{p}}{1-\hat{p}}$. Let $\hat{\mathbf{Q}}$ and $\hat{\mathbf{R}}$ denote two 2×2 matrices, the (i,j)-th elements of which being $\hat{q}_{i-1,j-1}$ and $\hat{r}_{i-1,j-1}$, respectively. Assuming that the cell counts for the cases and controls are distributed according to two different independent multinomial distributions, the asymptotic variance-covariance matrices are

$$\mathrm{Var}\hat{\mathbf{Q}}=\mathrm{Var}\left(\mathrm{vec}\hat{\mathbf{Q}}\right)=\frac{1}{N_1}\times\left[\mathrm{Diag}\left(\mathrm{vec}\hat{\mathbf{Q}}\right)-\left(\mathrm{vec}\hat{\mathbf{Q}}\right)\left(\mathrm{vec}\hat{\mathbf{Q}}\right)^{\mathbf{t}}\right]$$

$$\mathrm{Var}\hat{\mathbf{R}}=\mathrm{Var}\left(\mathrm{vec}\hat{\mathbf{R}}\right)=\frac{1}{N_0}\times\left[\mathrm{Diag}\left(\mathrm{vec}\hat{\mathbf{R}}\right)-\left(\mathrm{vec}\hat{\mathbf{R}}\right)\left(\mathrm{vec}\hat{\mathbf{R}}\right)^{\mathbf{t}}\right],$$

respectively.

Let $\hat{\mathbf{\Theta}}$ be a 2×2 matrix with its (i,j)-th element being $\frac{\hat{p}}{1-\hat{p}}\times\frac{\hat{q}_{i-1,j-1}}{\hat{r}_{i-1,j-1}}$. Using the delta method, we have that

$$\mathrm{Var}\hat{\mathbf{\Theta}}=\left(\frac{\partial\hat{\mathbf{\Theta}}}{\partial\hat{\mathbf{P}}}\right)^{\mathbf{t}}\left(\mathrm{Var}\hat{\mathbf{P}}\right)\left(\frac{\partial\hat{\mathbf{\Theta}}}{\partial\hat{\mathbf{P}}}\right)+\left(\frac{\partial\hat{\mathbf{\Theta}}}{\partial\hat{\mathbf{Q}}}\right)^{\mathbf{t}}\left(\mathrm{Var}\hat{\mathbf{Q}}\right)\left(\frac{\partial\hat{\mathbf{\Theta}}}{\partial\hat{\mathbf{Q}}}\right)+\left(\frac{\partial\hat{\mathbf{\Theta}}}{\partial\hat{\mathbf{R}}}\right)^{\mathbf{t}}\left(\mathrm{Var}\hat{\mathbf{R}}\right)\left(\frac{\partial\hat{\mathbf{\Theta}}}{\partial\hat{\mathbf{R}}}\right),$$

where $\frac{\partial\hat{\mathbf{\Theta}}}{\partial\hat{\mathbf{P}}}$, $\frac{\partial\hat{\mathbf{\Theta}}}{\partial\hat{\mathbf{Q}}}$, and $\frac{\partial\hat{\mathbf{\Theta}}}{\partial\hat{\mathbf{R}}}$ are 4×4 diagonal matrices, with the $(i+2\times j-2)$-th diagonal elements being $\frac{\hat{q}_{i-1,j-1}}{\hat{r}_{i-1,j-1}}$, $\frac{\hat{p}}{1-\hat{p}}\times\frac{1}{\hat{r}_{i-1,j-1}}$, and $-\frac{\hat{p}}{1-\hat{p}}\times\frac{\hat{q}_{i-1,j-1}}{\hat{r}_{i-1,j-1}^{\;2}}$, respectively, for $i,j\in\{1,2\}$, and that

$\mathrm{Var}\left(\log\widehat{\mathrm{PRISM}}\right)=\mathbf{C}\left(\mathrm{Var}\hat{\mathbf{\Theta}}\right)\mathbf{C}^{\mathbf{t}}$, where

$$\mathbf{C} = \left[\left(1 + \frac{\hat{p}}{1-\hat{p}} \times \frac{\hat{q}_{0,0}}{\hat{r}_{0,0}} \right)^{-1}, -\left(1 + \frac{\hat{p}}{1-\hat{p}} \times \frac{\hat{q}_{1,0}}{\hat{r}_{1,0}} \right)^{-1}, -\left(1 + \frac{\hat{p}}{1-\hat{p}} \times \frac{\hat{q}_{0,1}}{\hat{r}_{0,1}} \right)^{-1}, \left(1 + \frac{\hat{p}}{1-\hat{p}} \times \frac{\hat{q}_{1,1}}{\hat{r}_{1,1}} \right)^{-1} \right].$$

S2 Exhibit. R code for the proposed method.

```
##########################################################################

# n.case: a vector indicating the number of 'cases' for each exposure profile of        #

#            (X=1,Z=1), (X=1,Z=0), (X=0,Z=1), and (X=0,Z=0), respectively                #

# n.control: a vector indicating the number of 'controls' for each exposure profile    #

#                of (X=1,Z=1), (X=1,Z=0), (X=0,Z=1), and (X=0,Z=0), respectively        #

# prev: an estimate of the disease prevalence from vital statistics                      #

# n.pop: the population size of vital statistics (default value 100000)                  #

##########################################################################

n.case <- c(20,13,161,117)
n.control <- c(18,24,261,319)
prev <- 0.252
n.pop <- 100000

non.rare <- function( n.case, n.control, prev, n.pop=10^6){

    q.xz <- n.case/sum(n.case)
    r.xz <- n.control/sum(n.control)
    pre.odds <- prev/(1-prev)
    odds.xz <- pre.odds*(q.xz/r.xz)
    contrast <- c(1,-1,-1,1)
    logperil <- log(1+odds.xz)
    logPRISM <- sum(t(contrast)%*%logperil)

    n.dis <- n.pop*prev
    var.p <- matrix(rep(pre.odds^2*(1/n.dis+1/(n.pop-n.dis)),16),4,4)
    var.q <- (diag(c(q.xz))-(q.xz)%*%t(q.xz))/sum(n.case)
    var.r <- (diag(c(r.xz))-(r.xz)%*%t(r.xz))/sum(n.control)
```

```
    diff.p <- diag(c(q.xz/r.xz))
    diff.q <- diag(c(pre.odds/r.xz))
    diff.r <- diag(c(-odds.xz/r.xz))
    variance <- diff.p%*%var.p%*%diff.p+diff.q%*%var.q%*%diff.q+diff.r%*%var.r%*%diff.r
    diff <- (1/(1+odds.xz))*contrast
    varlogPRISM <- t(diff)%*%variance%*%diff
    z.statistics <- logPRISM^2/varlogPRISM        #z^2
    p.value <- 1-pchisq(z.statistics,1)
    return(p.value)

}

non.rare(n.case,n.control,prev,n.pop)
non.rare(n.case,n.control,prev)
```

S3 Exhibit. SAS code for the proposed method.

```
proc iml;

Case=
{
20 13      /** number of cases for X=1, the 1st column is for Z=1, 2nd for Z=0 **/
161 117    /** number of cases for X=0, the 1st column is for Z=1, 2nd for Z=0 **/
};
Control=
{
18 24      /** number of controls for X=1, the 1st column is for Z=1, 2nd for Z=0 **/
261 319    /** number of controls for X=0, the 1st column is for Z=1, 2nd for Z=0 **/
};
prevalence = 0.252; /** an estimate of the disease prevalence from vital statistics **/
n_pop = 10**6;    /** the population size of vital statistics (default value 100000) **/


n_dis = n_pop*prevalence;
q_xz = Case/Case[+];
r_xz = Control/Control[+];
pre_odds = prevalence/(1-prevalence);
odds_xz = pre_odds*(q_xz/r_xz);
contrast =
{
1 -1 -1 1
};
logperil = log(1+odds_xz);
logPRISM = contrast*logperil`;

pre_p = (pre_odds**2)*(1/n_dis+1/(n_pop-n_dis));
var_p = shape(pre_p,4,4);
var_q = ( Diag(q_xz) - q_xz` * q_xz ) /Case[+];
var_r = ( Diag(r_xz) - r_xz` * r_xz ) /Control[+];
diff_p = Diag(q_xz/r_xz);
diff_q = Diag(pre_odds/r_xz);
diff_r = Diag(-odds_xz/r_xz);
```

```
variance = diff_p*var_p*diff_p`+ diff_q*var_q*diff_q`+ diff_r*var_r*diff_r`;
diff = (1/(1+odds_xz))*Diag(contrast);
varlogPRISM = diff*variance*diff`;
z = (logPRISM*logPRISM)/varlogPRISM;
p_value = 1-probchi(z,1);

print p_value;
```

S4 Exhibit. The algorithm of the variance in the risk-scale RERI test

RERI is defined as $\mathrm{RERI} = \mathrm{RR}_{1,1} - \mathrm{RR}_{1,0} - \mathrm{RR}_{0,1} + 1$. A test of $\mathrm{RERI} > 1$ is equivalent to a test of $\mathrm{Risk}_{1,1} - \mathrm{Risk}_{1,0} - \mathrm{Risk}_{0,1} > 0$. For the risk-scale RERI test, we use $\hat{p}$ to estimate the disease risk as $\widehat{\mathrm{Risk}}_{x,z} = \dfrac{\widehat{\mathrm{Odds}}_{x,z}}{1+\widehat{\mathrm{Odds}}_{x,z}} = \dfrac{\dfrac{\hat{p}}{1-\hat{p}} \times \dfrac{\hat{q}_{x,z}}{\hat{r}_{x,z}}}{1+\dfrac{\hat{p}}{1-\hat{p}} \times \dfrac{\hat{q}_{x,z}}{\hat{r}_{x,z}}}$, and then we test

$$\hat{\delta} = \frac{\dfrac{\hat{p}}{1-\hat{p}} \times \dfrac{\hat{q}_{1,1}}{\hat{r}_{1,1}}}{1+\dfrac{\hat{p}}{1-\hat{p}} \times \dfrac{\hat{q}_{1,1}}{\hat{r}_{1,1}}} - \frac{\dfrac{\hat{p}}{1-\hat{p}} \times \dfrac{\hat{q}_{1,0}}{\hat{r}_{1,0}}}{1+\dfrac{\hat{p}}{1-\hat{p}} \times \dfrac{\hat{q}_{1,0}}{\hat{r}_{1,0}}} - \frac{\dfrac{\hat{p}}{1-\hat{p}} \times \dfrac{\hat{q}_{0,1}}{\hat{r}_{0,1}}}{1+\dfrac{\hat{p}}{1-\hat{p}} \times \dfrac{\hat{q}_{0,1}}{\hat{r}_{0,1}}} > 0.$$

Also, we have $\mathrm{Var}\left(\hat{\delta}\right) = \mathbf{E}\left(\mathrm{Var}\hat{\boldsymbol{\Theta}}\right)\mathbf{E}^{\mathrm{t}}$, where

$$\mathbf{E} = \left[0, -\left(1+\frac{\hat{p}}{1-\hat{p}} \times \frac{\hat{q}_{1,0}}{\hat{r}_{1,0}}\right)^{-2}, -\left(1+\frac{\hat{p}}{1-\hat{p}} \times \frac{\hat{q}_{0,1}}{\hat{r}_{0,1}}\right)^{-2}, \left(1+\frac{\hat{p}}{1-\hat{p}} \times \frac{\hat{q}_{1,1}}{\hat{r}_{1,1}}\right)^{-2}\right].$$

The details of $\mathrm{Var}\hat{\boldsymbol{\Theta}}$ is in S1 Exhibit. The risk-scale RERI test is a Z-test: $Z = \dfrac{\hat{\delta}}{\sqrt{\mathrm{Var}\left(\hat{\delta}\right)}}$, and a sufficient-cause interactions can be declared when the test statistics Z is in the rejection region.

S5 Exhibit. The powers of the proposed method when incorporating the disease prevalence from a previous study with different sample size.

The following figures show the powers of the proposed method when incorporating the disease prevalence from a previous study (with a sample size of 10^2, 5×10^2, 10^3, 10^4, 10^5, and 10^6, respectively). Assume that a case-control study recruited a total of 500 cases and 500 controls (Panel A, D, and G), 1000 cases and 1000 controls (Panel B, E, and H), and 5000 cases and 5000 controls (Panel C, F, and I), respectively. It can be seen that the sample size of the previous study can be as small as 1000 without excessively imparing the power.

(I) Assume that the relative risk for X is $\text{RR}_{1,0}=3$, and for Z, $\text{RR}_{0,1}=2$. We examined the powers under the alternative hypothesis, respectively, when the disease prevalence is 0.02 ($\text{PRISM}=1.0155$, 1.0109, and 1.0048, for Panel A, B and C, respectively), when it is 0.2 ($\text{PRISM}=1.1710$, 1.1172, and 1.0500 for Panel D, E, and F, respectively), and when it is 0.4 ($\text{PRISM}=1.4072$, 1.2620, and 1.1080 for Panel G, H, and I, respectively).

(II) Assume that the relative risk for X is $\text{RR}_{1,0}=5$, and for Z, $\text{RR}_{0,1}=4$. We examined the powers under the alternative hypothesis, respectively, when the disease prevalence is 0.02 ($\text{PRISM}=1.0160$, 1.0111, and 1.0049, for Panel A, B and C, respectively), when it is 0.2 ($\text{PRISM}=1.1780$, 1.1200, and 1.0520 for Panel D, E, and F, respectively), and when it is 0.4 ($\text{PRISM}=1.4220$, 1.2790, and 1.1132 for Panel G, H, and I, respectively).

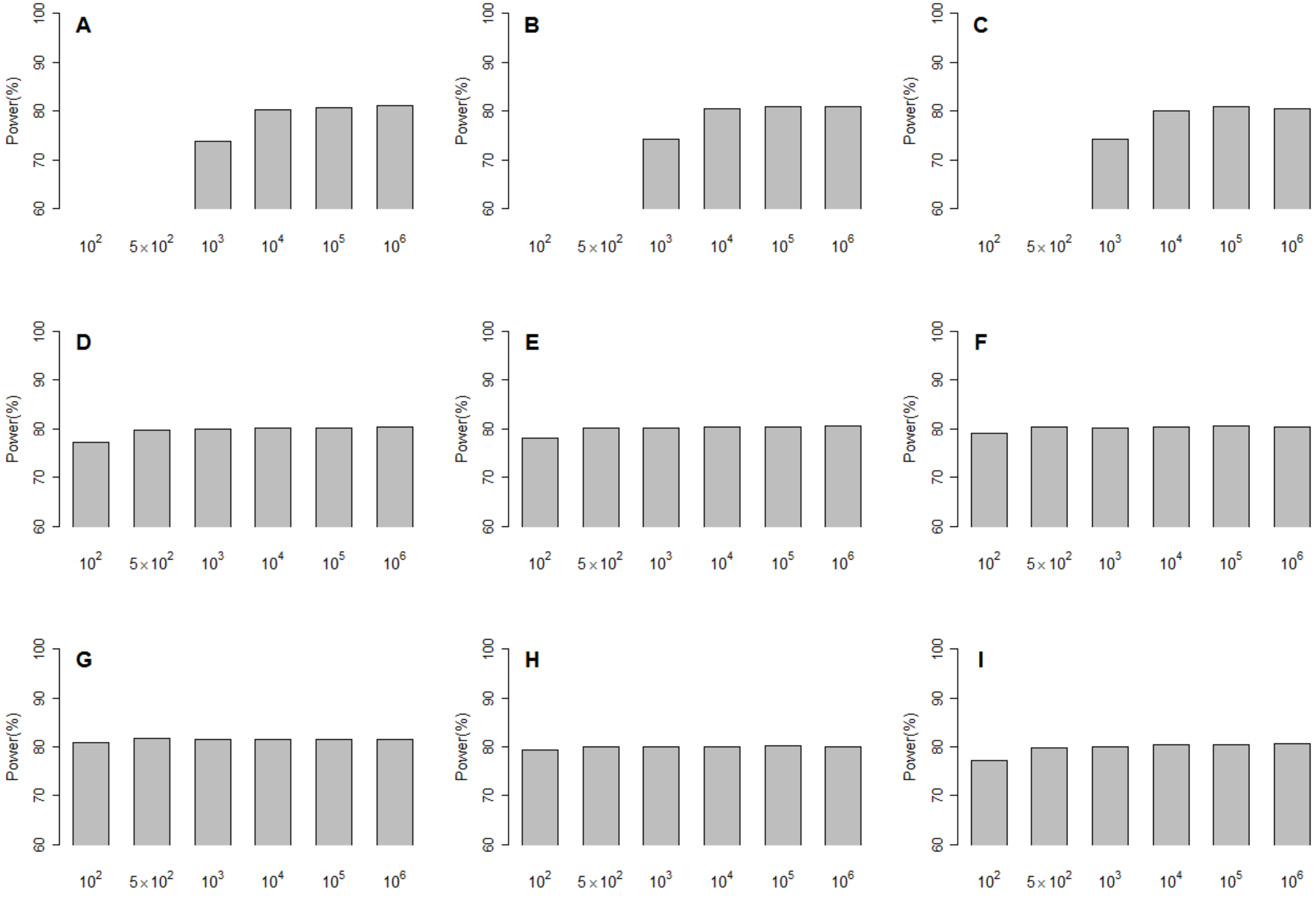
A
B
C
D
E
F
G
H
I
Power(%)
60
70
80
90
100
10^2
5×10^2
10^3
10^4
10^5
10^6

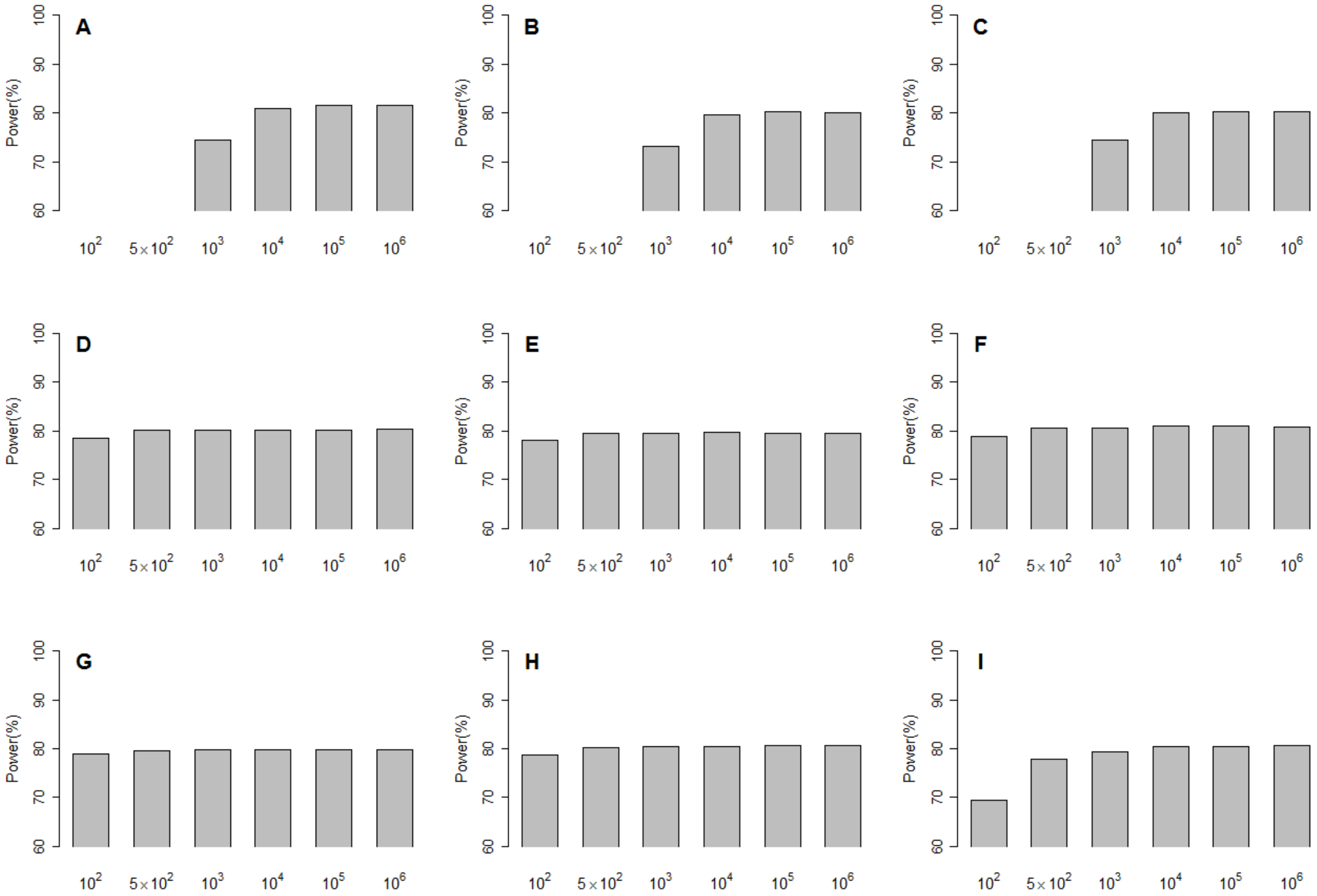
A
B
C
D
E
F
G
H
I
Power(%)
60
70
80
90
100
10^2
5×10^2
10^3
10^4
10^5
10^6
